# Supplementary material for: The genome sequence of a click beetle, Agrypnus murinus (Linnaeus, 1758)
Source: Wellcome Open Res. 2023 Aug 18;8:355. [Version 1] doi: 10.12688/wellcomeopenres.19847.1 (PMC11319899; doi:10.12688/wellcomeopenres.19847.1)
Supplement: Peer review history

# Reviewer report: Reviewer response for version 1

**DOI:** 10.21956/wellcomeopenres.21980.r92756

**Published:** 2024-08-23

**Authors:** Chelsea Skojec

**Affiliations:** 1 University of Florida, Gainesville, Florida, USA

In this manuscript entitled “The genome sequence of a click beetle, *Agrypnus murinus* (Linnaeus, 1758)” authors present a high-quality genome of the click beetle, *Agrypnus murinus. *A genome annotation for *A. murinus *will benefit future pest and soil studies, as it is a substantial agricultural pest species. The genome is high quality, with a reported genome assembly of 1578.9 Mb and a BUSCO completeness of 99.2%. The manuscript is clearly written, figures are clear and readable, the sequencing and bioinformatics methods are appropriate, using the current state of the art technologies and methods for extraction, sequencing and assembly.

Are sufficient details of methods and materials provided to allow replication by others?

Yes

Is the rationale for creating the dataset(s) clearly described?

Yes

Are the datasets clearly presented in a useable and accessible format?

Yes

Are the protocols appropriate and is the work technically sound?

Yes

Reviewer Expertise:

Genomics, genetics, phylogenetics, bioinformatics, entomology.

I confirm that I have read this submission and believe that I have an appropriate level of expertise to confirm that it is of an acceptable scientific standard.

# Reviewer report: Reviewer response for version 1

**DOI:** 10.21956/wellcomeopenres.21980.r92755

**Published:** 2024-08-12

**Authors:** Yuhao Huang

**Affiliations:** 1 Sun Yat-sen University, Guangzhou, China

The data note presents a high-quality, chromosome-level genome assembly of the click beetle *Agrypnus murinus*, with a completeness of 99.2%. This genome assembly will be valuable for understanding this agricultural pest and for research into genetic diversity.

 The rationale for creating the dataset is well-justified. The sequencing and assembly methods employed are both reasonable and reproducible. Additionally, the raw data, assembly, and annotations are accessible.

 I approve of the manuscript in its current form. However, there are a few comments that could enhance the manuscript and the dataset for user convenience:

1. Although the link to the annotations is provided in the genome annotation report section, it is recommended to also include this link in the data availability section to improve visibility.
2. In the mitochondrial genome assembly method, "MitoFinder or MITOS" was used for the annotation. Please clarify which tool was used for this species. In addition, it would be beneficial to provide the mitochondrial genome annotation as well.
3. The predicted gene count is 42,204, which seems unusually high based on my experience with beetle genomes, unless a large-scale duplication has occurred in this species. This high number might result from fragmented gene predictions by BRAKER2, particularly if only the protein mode was utilized. I have noticed transcriptomic sequencing data under BioProject accession PRJEB46297. A more refined version of gene predictions using transcriptomic data and the complete Ensembl pipeline would be advantageous for users of this genome.

Are sufficient details of methods and materials provided to allow replication by others?

Yes

Is the rationale for creating the dataset(s) clearly described?

Yes

Are the datasets clearly presented in a useable and accessible format?

Yes

Are the protocols appropriate and is the work technically sound?

Yes

Reviewer Expertise:

comparative genomics, evolutionary biology, bioinformatics, phylogenetics, beetle

I confirm that I have read this submission and believe that I have an appropriate level of expertise to confirm that it is of an acceptable scientific standard.

# Reviewer report: Reviewer response for version 1

**DOI:** 10.21956/wellcomeopenres.21980.r85302

**Published:** 2024-06-11

**Authors:** R Axel W Wiberg

**Affiliations:** 1 Stockholm University, Stockholm, Sweden

The Data Note describes the genome assembly of beetle species *Agrypnus murinus* using the DTOL standardised pipeline. Overall I think the manuscript is very good.

 The rationale for creating the dataset is well described. Although I think there could be some important additions here (see below).

 It is technically sound with the methods/protocols well described and referenced.

 The references to the DTOL nextflow pipelines and other software used ensure that replication would be straightforward.

 The results and datasets are mostly clearly presented (see below). The genome sequence and the annotation are easily available.

 I think the article is valid in it's current form, but do have a few additional comments:

**1)** In the 1 ^st^ paragraph of the “Background” section the authors write:

 “Adults grow up to 17 mm in length..”

 There is often a sexual dimorphism in insects, I would like to see sizes for males and females separately, or if there is no sexual dimorphism for that to be stated.

**2)** Coleopterans vary a lot in their sex-determination systems and sex-chromosome karyology. While most are XY, XO systems are common in several groups. For this species, and especially because the specimen sequenced here is male, it would be really useful to note in the text that, while the karyology of *Agrypnus murinus *seems unknown at the moment, other close relatives are XO. Indeed, among the Elateridae, XO systems reach nearly 70% of examined species. Thus, a Y chromosome is not expected in the assembly. Adding this information would add context to those using the genome, increase it the generality of the note.

 See the very useful database here: http://coleoguy.github.io/karyotypes/

 and the citations here:

 [ ref 1 ]

 [ ref 2 ]

 In addition, the data note mentions 8 autosomes and the X chromosome in the assembly. Is the general karyology of the species well known? Are 8 autosomes expected, I’m not sure if there is information on the full karyotype of the species?

**3** **)** The ~42,000 genes seems on the high end from my experience. In another Coleopteran genome that I was involved in, annotation was quite fragmented at least in part due to the high number of repetitive elements. I think it would be useful to include in this Data Note (and perhaps in DTOL’s standardised pipelines in general) a simple RepeatModeler and RepeatMasker analysis to get at least a rough picture of the repeat content.

 RepeatModeler: https://www.repeatmasker.org/RepeatModeler/

 RepeatMasker: https://www.repeatmasker.org/

 It is in general a useful thing for a genome assembly to have as it helps in all sorts of down-stream applications.

**4)** Figure 5 would benefit from some kind of x- and y- axis labels at least for the largest scaffolds. Which is the X chromosome? One can work it out from table 2, but axis labels would be better I think.

**5) **As far as I can tell, the mtGenome is present as a contig in the assembly file, but the annotation files do not include the mtGenome. Would it be possible to integrate the MITOS annotation somehow? Or provide it separately? This would make the resource much more accessible and useful.

Are sufficient details of methods and materials provided to allow replication by others?

Yes

Is the rationale for creating the dataset(s) clearly described?

Partly

Are the datasets clearly presented in a useable and accessible format?

Partly

Are the protocols appropriate and is the work technically sound?

Yes

Reviewer Expertise:

evolutionary biology, comparative and population genomics, sexual selection and sexual conflict

I confirm that I have read this submission and believe that I have an appropriate level of expertise to confirm that it is of an acceptable scientific standard.

## References

[1] Reference source

[2] Reference source
